# Influence of training level on cervical cone size and resection margin status at conization: a retrospective study

**DOI:** 10.1007/s00404-018-4761-1

**Published:** 2018-03-30

**Authors:** Eliana Montanari, Christoph Grimm, Richard Schwameis, Lorenz Kuessel, Stephan Polterauer, Chiara Paternostro, Heinrich Husslein

**Affiliations:** 1 Department of Gynecology and Obstetrics, Medical University of Vienna, Waehringer Guertel 18-20, 1090 Vienna, Austria; 2 Medical University of Vienna, Waehringer Guertel 18-20, 1090 Vienna, Austria

**Keywords:** Cervical conization, LLETZ, Surgical training, CIN, Incomplete resection

## Abstract

**Objective:**

To explore whether a surgeon’s training level influences the rate of incomplete resections or the amount of resected cervical tissue in women treated with large loop excision of the transformation zone (LLETZ).

**Methods:**

The present study is a retrospective analysis of the data of women who had undergone LLETZ for cervical intraepithelial neoplasia (CIN) within the years 2004–2008 at the Medical University of Vienna. Women were grouped according to the level of training of the operating surgeon (i.e, resident or staff gynecologist) and univariate and multivariable analyses were performed to identify independent risk factors for excessive cone volume, depth and incomplete resection (i.e., positive resection margin).

**Results:**

Data of 912 women were analysed. Residents had a significantly larger cone volume [median 2681 (interquartile range 1472–4109) mm^3^] than staff gynecologists [2094 (1309–3402) mm^3^] (*p* = 0.001) in univariate analysis. The depth of resection and the rate of incomplete resection were comparable between both groups. In a binary logistic multivariable analysis, the level of training as well as patient’s age was significantly associated with a cone volume larger than 2500 mm^3^.

**Conclusion:**

Conization performed by residents as opposed to staff gynecologists does not compromise the procedure’s effectiveness but may expose women to a potential additional risk for adverse obstetrical outcomes due to excessive resection of cervical tissue.

**Electronic supplementary material:**

The online version of this article (10.1007/s00404-018-4761-1) contains supplementary material, which is available to authorized users.

## Introduction

Over the past decade, there has been growing interest in the association between treatment for cervical intraepithelial neoplasia (CIN) and subsequent adverse obstetrical outcome. CIN, especially grades II and III, is nowadays most commonly treated by large loop excision of the transformation zone (LLETZ) [1]. The procedure is considered successful if the lesion, including the entire transformation zone, is completely resected. This should be achieved using the smallest possible cone size. Incomplete resection (i.e., positive resection margin) often requires further treatment and/or repeat surgery resulting in additional removal of cervical tissue [2–5].

Several studies, including meta-analyses, have demonstrated the increased risk of adverse pregnancy outcomes such as preterm birth, low birthweight, and perinatal mortality following LLETZ and other surgical treatment options such as cold knife or laser conization [6–10]. In this context, the risk of spontaneous preterm delivery seems to increase with the amount of cervical tissue removed. Both a cone volume larger than 2500 mm^3^ and a depth of the excised surgical specimen ≥ 20 mm seem to be associated with a higher rate of preterm birth [6, 9–12].

Large loop excision of the transformation zone represents a procedure typically carried out early in gynecologic residency training, because it is considered to be a minor surgical procedure and major intraoperative complications are uncommon [13]. Nevertheless, this procedure can be difficult to teach and it has been shown that competency requires deliberate practice [14]. As with any other surgical procedure, the acquisition of competency performing LLETZ follows a learning curve for each surgeon [15, 16]. It can be hypothesized that experienced surgeons are able to adapt cone size individually to lesion factors (e.g., size and location) as well as patient factors (age and fertility issues). Less experienced surgeons may not be able to adapt cone size individually and may excise more cervical tissue than necessary, because they are afraid of not removing the entire lesion. On the other hand, they may be afraid of removing excessive amounts of cervical tissue, resulting in incomplete resection, potentially necessitating a second surgery.

Therefore, the aim of the present retrospective study was to explore whether the surgeon’s level of training has an effect on the rate of incomplete resections (positive resection margins of the surgical specimens) and/or the amount of cervical tissue resected during LLETZ.

## Materials and methods

### Study design

This study is a retrospective analysis of a prospectively generated database including all consecutive women who had undergone LLETZ for cervical dysplasia between 2004 and 2008 at the Department of Gynecology and Obstetrics of the Medical University of Vienna. Exclusion criteria were re-conization, treatment by techniques other than LLETZ and missing information about cone dimensions. The study was approved by the Ethics Committee of the Medical University of Vienna (EK number 1712/2015, approved September 2015). According to the University Ethics Committee, a formal consent was not required for this type of study, and therefore, the need for informed consent for participation was waived.

The following parameters were abstracted from the database: woman’s age, level of training of the operating surgeon (i.e., resident or staff gynecologist with an additional subdivision of residents into those with up to 12 months vs those with more than 12 months of gynecologic rotations during residency), cone dimensions (height, width and depth), resection margin status for overall, endocervical and ectocervical margins, preoperative histology of colposcopy guided cervical biopsies, preoperative Pap smear, preoperative human papillomavirus (HPV) status, Body Mass Index (BMI) and smoking status. All residents performed the conizations under supervision of a staff gynecologist who was present at the time of surgery. The same technique was used for all conizations. Loop size was chosen according to the preoperative colposcopy report and after intraoperative application of diluted Lugol solution to the cervix. The smallest loop to allow a complete resection was chosen. Conization was performed under direct visualization without the use of a colposcope.

### Outcome measures

Assuming the surgical specimen obtained by LLETZ to resemble a hemiellipsoid, the cone volume was calculated using the formula (1/2) × (4/3) × *π* × (length/2) × (width/2) × depth as previously described [17]. In cases where two cones were resected, or the cone was resected in several pieces, the volumes were added. The primary outcome measures were conization depth and volume as well as resection margin status of surgical specimens obtained by gynecology residents compared to those taken by staff gynecologists. In addition, a subgroup analysis was performed to assess whether conization depth and volume as well as resection margin status differed between residents at the beginning of their residency compared to more experienced residents.

### Statistical analysis

Data were analysed by descriptive statistics. Woman’s age, BMI and volume, and depth of the excised cones were compared between residents and staff gynecologists as well as between residents having already had more or less than 12 months of gynecologic rotation during residency using the Mann–Whitney *U* test. Cone volumes were divided using the cut-off value of 2500 mm^3^ and compared between groups by the Chi-squared test. Depths of cones were also divided into groups (with cut-off values of 10, 15, or 20 mm, respectively), and compared between residents and staff gynecologists (and between the two resident subgroups, respectively) by the Chi-squared test. Possible associations between the surgeon’s level of training and other characteristics, i.e., resection margin status, preoperative histology of colposcopy guided cervical biopsies, preoperative Pap smear, presence of high risk HPV and smoking status, were analysed by Chi-squared tests. Two-sided *p* values < 0.05 were considered statistically significant. A binary logistic multivariable regression model was fit to evaluate the association between a gynecologist’s level of training and an excised cone volume greater than 2500 mm^3^ together with the woman’s age. IBM SPSS version 21.0 (IBM Corp., Armonk, NY, USA) was used for data analysis.

## Results

Between 2004 and 2008, 1041 conization procedures were performed at our department. Due to missing information about cone dimensions, use of techniques other than LLETZ or the operation being a re-conization, 129 women (12%) were excluded from the study. A total of 912 women were included in the final analysis. Women’s characteristics for both the resident and the staff gynecologist study group are provided in Table 1. No significant differences regarding women’s age, preoperative cytology, preoperative HPV status, or preoperative histology of colposcopically guided biopsies were observed between groups (Table 1). BMI was significantly higher in the residents than in the staff gynecologist group and more smokers could be found in the residents than in the staff gynecologist group (Table 1).

**Table 1:** Characteristics of patients undergoing conization performed by residents and staff gynecologists

Patients’ characteristics	Residents (*n* = 341)	Staff gynecologists (*n* = 571)	*p* value
Age (years), median (IQR)	34 (29–41)	34 (29–40)	0.734
BMI (kg/m^2^), median (IQR)	23 (21–26)	22 (20–24)	0.002
Smoker			0.037
Yes	167 (49%)	242 (42%)	
No	120 (35%)	238 (42%)	
Unknown	54 (16%)	91 (16%)	
Preoperative histology			0.185
CIN2	124 (36%)	156 (27%)	
CIN3	153 (45%)	182 (32%)	
Carcinoma	0 (0%)	4 (1%)	
Unknown	64 (19%)	229 (40%)	
Preoperative Pap smear			0.544
LSIL	139 (41%)	178 (31%)	
HSIL	187 (55%)	262 (46%)	
Unknown	15 (4%)	131 (23%)	
Preoperative HPV status			0.144
High risk negative	8 (2.3%)	18 (3.15%)	
High risk positive	267 (78.3%)	322 (56.39%)	
Unknown	66 (19.4%)	231 (40.46%)	

Patient’s age and BMI were compared between groups using the Mann–Whitney *U* test and are shown as median (IQR). All the other data were analysed by the Chi-squared test and are shown as *n* (% within the group)

*IQR* interquartile range, *BMI* Body Mass Index, *CIN* cervical intraepithelial neoplasia, *LSIL* low-grade squamous intraepithelial lesion, *HSIL* high-grade SIL, *HPV* human papilloma virus

There was no statistically significant difference in the depths of the excised cone specimens between the resident and the staff gynecologist group (Table 2). After classification of the depth of excision according to cut-off values of 10, 15, or 20 mm, still no significant difference was found between groups (Table 2). With regard to the proportion of positive resection margins, no statistically significant difference could be observed between groups (Table 2). The volumes of the excised cones differed significantly between residents and staff gynecologists (Table 2) with higher values in the residents group. This difference was also present when cone volume was categorised according to a cut-off value of 2500 mm^3^, showing a significantly higher percentage of cone volumes greater than this value in the residents group compared to the staff gynecologist group (54% of all conizations performed in the residents group compared to 42% of all conizations performed in the staff gynecologist group, *p* < 0.001, Table 2).

**Table 2:** Comparison of large loop excision of the transformation zone (LLETZ) findings between conizations performed by residents and staff gynecologists

Conization data	Residents (*n* = 341)	Staff gynecologists (*n* = 571)	*p* value
Cone volume (mm^3^), median (IQR)	2680 (1472–4109)	2094 (1309–3402)	0.001
Cone volume > 2500 mm^3^			< 0.001
Yes	183 (54%)	237 (41.5%)	
No	158 (46%)	334 (58.5%)	
Cone depth (mm), median (IQR)	15 (10–19)	14 (10–18)	0.186
Cone depth > 10 mm			0.162
Yes	253 (74%)	399 (70%)	
No	88 (26%)	172 (30%)	
Cone depth > 15 mm			0.102
Yes	144 (42%)	210 (37%)	
No	197 (58%)	361 (63%)	
Cone depth > 20 mm			0.422
Yes	50 (15%)	73 (13%)	
No	291 (85%)	498 (87%)	
Positive cone margin (overall)			0.599
Yes	63 (18%)	114 (20%)	
No	275 (81%)	454 (79.5%)	
Unknown	3 (1%)	3 (0.5%)	
Positive ectocervical margin			0.315
Yes	39 (11%)	79 (13.8%)	
No	299 (88%)	491 (86.0%)	
Unknown	3 (1%)	1 (0.2%)	
Positive endocervical margin			0.507
Yes	35 (10%)	67 (11.7%)	
No	303 (89%)	501 (87.7%)	
Unknown	3 (1%)	3 (0.5%)	

Continuous data were compared between groups using the Mann–Whitney *U* test and are shown as median (IQR). Nominal data were analysed by the Chi-squared test and are shown as *n* (% within the group)

*IQR* interquartile range

In addition, a binary logistic multivariable regression model including woman’s age and the level of training of the operating surgeon was performed. In this multivariable model, both woman’s age and level of training were significantly associated with a cone volume larger than 2500 mm^3^ (Table 3).

**Table 3:** Binary logistic multivariable analysis for a cone volume greater than 2500 mm^3^

Variable	OR (95% CI)	*p* value
Level of training (residents vs staff gynecologists)	1.65 (1.257–2.166)	< 0.001
Patient’s age (per year)	1.025 (1.011–1.040)	0.001

*OR (95% CI)* odds ratio (95% confidence interval)

In a subgroup analysis comparing women treated by residents with more or less than 12 months of gynecologic rotations during residency training, no significant differences could be found regarding patient characteristics (Supporting Information Table 1), resection margin status, or volume and depth of the excised cones (Table 4).

**Table 4:** Comparison of large loop excision of the transformation zone (LLETZ) findings between residents with ≤ 12 months of previous gynecologic rotation and residents with > 12 months of previous gynecologic rotation

Conization data	≤ 12 months of gynecologic rotation (*n* = 154)	> 12 months of gynecologic rotation (*n* = 187)	*p* value
Cone volume (mm^3^), median (IQR)	2779 (1490–4201)	2396 (1437–3927)	0.403
Cone volume > 2500 mm^3^			0.068
Yes	91 (59%)	92 (49%)	
No	63 (41%)	95 (51%)	
Cone depth (mm), median (IQR)	14.5 (10–19)	15 (10–18)	0.665
Cone depth > 10 mm			0.854
Yes	115 (75%)	138 (74%)	
No	39 (25%)	49 (26%)	
Cone depth > 15 mm			0.654
Yes	63 (41%)	81 (43%)	
No	91 (59%)	106 (57%)	
Cone depth > 20 mm			0.095
Yes	28 (18%)	22 (12%)	
No	126 (82%)	165 (88%)	
Positive cone margin (overall)			0.884
Yes	28 (18%)	35 (19%)	
No	125 (81%)	150 (80%)	
Unknown	1 (1%)	2 (1%)	
Positive ectocervical margin			0.572
Yes	16 (10%)	23 (12%)	
No	137 (89%)	162 (87%)	
Unknown	1 (1%)	2 (1%)	
Positive endocervical margin			0.926
Yes	16 (10.4%)	19 (10%)	
No	136 (88.3%)	167 (89%)	
Unknown	2 (1.3%)	1 (1%)	

Continuous data were compared between groups using the Mann–Whitney *U* test and are shown as median (IQR). Nominal data were analysed by the Chi-squared test and are shown as *n* (% within the group)

*IQR* interquartile range

## Discussion

### Main findings

In this retrospective, single center study, the level of training of the operating surgeon represented an independent risk factor for a greater amount of cervical tissue removed at the time of LLETZ. In contrast, treatment by residents did not compromise women’s safety with respect to the course of the disease, as there were no differences in the rate of incomplete resections (i.e., positive resection margins) compared to staff gynecologists. In univariate analysis, the cone volume excised by residents compared to staff gynecologists was observed to be significantly larger. In addition, the percentage of surgical specimens larger than 2500 mm^3^ was higher in the resident group compared to the staff gynecologist group. When level of training was evaluated in a binary logistic multivariable regression analysis together with the age of the woman, it remained an independent risk factor for cone volumes greater than 2500 mm^3^. In contrast, comparison of the cone depths showed no significant differences between the groups, neither concerning the depth nor the percentage of depths greater than 10, 15, or 20 mm, respectively. In a subgroup analysis of the resident cohort, there was no statistically significant difference of the volume and depth of the excised cones between more experienced compared to less experienced residents.

### Strengths and limitations

To our knowledge, this is the first study to explore whether the surgeon’s level of training has an effect on the rate of incomplete resections and/or the amount of cervical tissue resected during LLETZ. A considerable strength of this study is the large sample size and the prospective data collection over a 4 year period. Furthermore, the number of residents and staff gynecologists performing conization was high, increasing the external validity of our results.

The main limitation of this study is the secondary, retrospective data analysis, with all its inherent risks of bias. For example, data on preoperative histology were missing in 32% of cases. The vast majority of these women had a biopsy performed outside of the hospital and had to be labelled “unknown” because the histologic report was not available for this retrospective study due to limitations of the medical health care documentation system used during the study period. Furthermore, we were not able to assess the proportion of the excised amount of tissue in relation with the total cervical volume of the respective woman, since no data regarding total pre- and postoperative cervical volume were available. In a prospective study comparing pre- and postoperative cervical lengths using magnetic resonance tomography and transvaginal ultrasonography, the authors hypothesized that the proportion of the total resection volume could have a greater influence on the risk of premature birth than the depth of the excision cone [7]. However, as mentioned above, various studies found a link between the depth and volume of the excised cones with the risk of premature delivery irrespective of the total cervical volume. Therefore, comparison of these measures may nevertheless provide a good estimation of possible clinically relevant differences concerning treatment by residents compared to staff gynecologists.

### Interpretation

Over the last decades, the incidence of CIN in young women has been increasing, as has the average age at which women give birth [18]. As a result, a growing number of women has CIN prior to their first pregnancy. Cervical conization is the standard treatment for high-grade CIN [6–8]. The risk of adverse pregnancy outcome increases with the amount of cervical tissue removed. A recent study found both a cone volume larger than 2500 mm^3^ or a depth of the excised surgical specimen greater than 20 mm to be associated with a higher rate of preterm birth [11]. In the previous studies, a depth of the cone greater than 10 mm was shown to be linked to a significant increase in the risk of premature rupture of the membranes as well as in the risk of preterm delivery [6, 9]. Another group obtained similar findings regarding the depth of the removed cone, with women having received a medium (10–14 mm), large (15–19 mm), or very large (≥ 20 mm) excision showing a higher risk of preterm delivery than those with a small (< 10 mm) excision [12]. As for the cone size, in the same study, a total volume greater than 2660 mm^3^ was found to double the risk of preterm and very preterm delivery [12]. Other factors increasing the risk of adverse pregnancy outcome in women with treatment for CIN, such as a defective cervical antimicrobial barrier or decreased mechanical stability of the regenerated cervix, have been proposed [10]. However, reliable data are only available for the amount and dimensions of cervical tissue removed during conization.

As the volume and depth of the cone are ideally only as large as needed for a resection of the lesion with clear resection margins, it seems likely that there is a learning curve for the performance of this procedure.

In this study, LLETZ for the treatment of high-grade CIN performed by residents supervised by a staff physician resulted in the removal of a greater amount of cervical tissue compared to this procedure carried out by staff gynecologists and, therefore, may expose women to an additional risk for adverse obstetrical outcomes. An additional subgroup analysis revealed no statistically significant difference between procedures performed by more experienced compared to less experienced residents, which might be due to the limited case load performed during residency. The depth of the excised cone, another putative risk factor for adverse obstetrical outcomes, was not different in specimens obtained by residents compared to those obtained by staff gynecologists. Nevertheless, these findings underscore the importance of letting residents train on simulation models before practicing on real patients, as it is already recommended for many other surgical procedures [19]. Studies have consistently shown that skills acquired in the simulation-environment transfer to the operating room and that simulation-training programs significantly decrease the clinical learning curve of various operative procedures [20, 21]. Several inexpensive, easily constructed simulation models for conization are available [14, 22] that may help to get more confident with the technique and achieve competency through deliberate practice, thereby extending the limited case load performed during residency training.

Next to cone size and volume, the training level of the operating surgeon may also affect the rate of positive resection margins, thereby influencing the course of the disease and possibly making further treatment and/or repeat surgery necessary [2–5]. Only one retrospective study has addressed this question, showing that high volume surgeons achieved a higher rate of clear resection margins compared to residents and low volume staff members; however, there was no significant difference between residents and staff members [13]. In line with these findings, in our study no significant difference was found in the rate of positive resection margins between supervised residents and staff gynecologists, neither regarding the overall resection margin status nor ecto- or endocervical margins evaluated separately. Furthermore, the overall rate of incomplete resection in this study was < 20%, which is acceptably low and comparable to other studies [23]. Thus, performance of cervical conization by residents under supervision of a staff gynecologist seems to be safe for the patient with regard to the course of disease.

## Conclusion

In this retrospective, single center study, the level of training of the operating surgeon did not negatively affect the depth of the excised cone or the rate of incomplete resections (i.e., positive resection margins), but significantly influenced the total amount of cervical tissue removed at the time of LLETZ. Further studies including different residency programs are warranted to confirm our findings. In any case, this study underlines that simulation training of surgical procedures, even if they appear simple, should be promoted and should constitute an integral part of residency training.

## Electronic supplementary material

Below is the link to the electronic supplementary material.

Supplementary material 1 (DOC 51 kb)
