# Supplementary material for: Influence of training level on cervical cone size and resection margin status at conization: a retrospective study
Source: Arch Gynecol Obstet. 2018 Mar 30;297(6):1517–23. doi: 10.1007/s00404-018-4761-1 (PMC5945722; doi:10.1007/s00404-018-4761-1)
Supplement: Supplementary file 1 — Supplementary material 1 (DOC 51 kb) [file 404_2018_4761_MOESM1_ESM.doc]

**Supplemental Table 1.** Characteristics of patients undergoing conization performed by residents with ≤ 12 months of previous gynecologic rotation compared to residents with > 12 months of previous gynecologic rotation.

| Patients’ characteristics | ≤ 12 months of gynecologic rotation  (n=154) | > 12 months of gynecologic rotation  (n=187) | p-value |
| --- | --- | --- | --- |
| Age [years], median (IQR) | 36 (29-41) | 34 (29-41) | 0.420 |
| BMI [kg/m²], median (IQR) | 23 (21-26) | 23 (20-25) | 0.702 |
| Smoker  Yes  No  Unknown | 79 (51%)  57 (37%)  18 (12%) | 88 (47%)  63 (34%)  36 (19%) | 0.974 |
| Preoperative histology  CIN2  CIN3  Carcinoma  Unknown | 62 (40%)  62 (40%)  0 (0%)  30 (20%) | 62 (33%)  91 (49%)  0 (0%)  34 (18%) | 0.115 |
| Preoperative Pap smear  LSIL  HSIL  Unknown | 66 (42.9%)  81 (52.6%)  7 (4.5%) | 73 (39%)  106 (57%)  8 (4%) | 0.455 |
| Preoperative HPV status  High risk negative  High risk positive  Unknown | 4 (3%)  110 (71%)  40 (26%) | 4 (2%)  157 (84%)  26 (14%) | 0.619 |

IQR - interquartile range; BMI - body mass index; CIN - cervical intraepithelial neoplasia; LSIL - low grade squamous intraepithelial lesion; HSIL - high grade SIL; HPV - human papilloma virus. Patient's age and BMI were compared between groups using the Mann-Whitney-U test and are shown as median (IQR). All the other data were analysed by the chi-squared test and are shown as n (% within the group).
